# Measurement of and Factors Associated with the Anterior Chamber Volume in Healthy Chinese Adults

**DOI:** 10.1155/2017/6762047

**Published:** 2017-01-10

**Authors:** Yuan Zong, Qian Xu, Chunhui Jiang, Haohao Zhu, Jian Yu, Xinghuai Sun

**Affiliations:** ^1^Department of Ophthalmology and Vision Science, Eye and ENT Hospital, Fudan University, Shanghai 200031, China; ^2^Key Laboratory of Myopia of State Health Ministry and Key Laboratory of Visual Impairment and Restoration of Shanghai, Shanghai 200031, China; ^3^Department of Ophthalmology, The Central Hospital of Zaozhuang Mining Group, Zaozhuang 277800, China; ^4^Department of Ophthalmology, People's Hospital of Shanghai, No. 5, Shanghai 200240, China

## Abstract

*Purpose*. To measure the anterior chamber volume (ACV) and determine factors associated with the ACV in healthy Chinese adults.* Methods*. In this cross-sectional study, we used swept-source optical coherence tomography (SS-OCT) to measure ACV and other anterior segment parameters. Factors associated with ACV were also determined.* Results*. A total of 313 healthy Chinese adults were enrolled. The anterior segment parameters, including ACV, could be measured by SS-OCT with excellent repeatability and reproducibility. There was a significant difference between the horizontal and vertical anterior chamber widths (ACW) (*P* < 0.05), with a mean difference of 390 *μ*m. The ACV (mean 153.83 ± 32.42 mm^3^) was correlated with most of the anterior segment parameters, especially anterior chamber depth (ACD), which accounted for about 85% of the variation of ACV. Most of the anterior segment parameters were significantly correlated with age, and the relative changes in ACV and ACD were greatest in subjects aged 41–50 years.* Conclusion*. ACV was correlated with most of the anterior segment parameters measured in this study, particularly ACD. The relatively large difference between horizontal and vertical ACW suggests that the ACV could and should be measured using multiple OCT scans.

## 1. Introduction

The anterior segment parameters, for example, the anterior chamber volume (ACV), are of significant interest to ophthalmologists [1, 2], especially glaucoma specialists. This is because ACV is closely associated with primary angle closure glaucoma (PACG), which is often characterized by a shallow and small anterior chamber [3–5]. Although PACG can lead to severe visual impairment or blindness, its progression can usually be stopped by early detection and appropriate interventions [6, 7]. An objective and reliable method of monitoring the anterior segment is of particular importance in the detection and follow-up of PACG.

Optic coherence tomography (OCT) can be used for noncontact, objective measurements of the anterior segment [8], including the ACV [9]. In most of the earlier studies, ACV was measured on a single OCT image [3, 10, 11]. But because the eye is not perfectly spherical, the measured parameters might be influenced by the measurement plane. Swept-source OCT (SS-OCT) systems can acquire a series of high-resolution OCT scans of the anterior segment within several seconds, and the ACV and other volumes of the anterior chamber can be calculated using four or more OCT scans [12].

The aims of the present study were to measure anterior segment parameters by SS-OCT in a relatively large cohort of healthy Chinese adults, to investigate the variability of ACV, and to determine the factors associated with ACV.

## 2. Subjects and Methods

### 2.1. Subjects

Healthy Chinese adults ≥ 18 years old were enrolled and underwent thorough ophthalmic evaluations, which included the following: best-corrected visual acuity (BCVA); refraction measured by an autorefraction system; calculation of the spherical equivalent (SE) as the spherical dioptre (D) plus one-half of the cylindrical dioptric power; measurement of intraocular pressure (IOP) by a noncontact tonometer (Topcon CT-80A Computerized Tonometer; Topcon, Tokyo, Japan); measurement of axial lengths (AL) using IOLmaster (version 3.01; Carl Zeiss Meditec, Jena, Germany), slit-lamp biomicroscopy; and direct ophthalmoscopic examination of the undilated fundus. Inclusion criteria for this study were BCVA ≥ 16/20, IOP ≤ 21 mmHg, AL of 21–25 mm, and SE between −3 and +1 D. The exclusion criteria were as follows: history of intraocular disease, ocular surgery including laser peripheral iridotomy, or trauma; BCVA < 16/20; AL of <21 mm or >25 mm; IOP > 21 mmHg; and family history of glaucoma in a first-degree relative. This study was approved by the Ethics Review Committee at Shanghai Eye, Ear, Nose and Throat Hospital, China. All subjects provided written informed consent and the study was performed in accordance with the tenets of the Declaration of Helsinki.

### 2.2. SS-OCT Data Acquisition and Processing

A commercially available SS-OCT system (CASIA SS-1000; Tomey Corporation, Nagoya, Japan; software version 6H.4) was used and all scans were obtained without pupil dilation in a well-lit room (340 lux) by a single observer (Y. Z.). During the examination, the subjects were asked to keep their head still and to stare at an internal target. The eyelids were opened gently by another technician to expose the upper and lower limbus. A three-dimensional scan of the anterior segment with 128 radial slices (each being 6 mm in depth and 16 mm in length) was obtained. For all subjects, ACV was automatically calculated using the volume protocol from 8 slices: 0°–180°, 23°–203°, 45°–225°, 68°–248°, 90°–270°, 113°–293°, 135°–315°, and 158°–338°. The cornea and iris boundaries were automatically detected by the system's software and were checked by an observer who was blinded to the subject's characteristics. If the software could not correctly detect the boundaries, they were manually adjusted. The ACV and iris volume (IV) were automatically calculated using the system's software and the pupil diameter (PD) was also determined. The ACD and anterior chamber width (ACW) were measured using the horizontal (0°–180°) and vertical (90°–270°) slices. After manually locating the angle recesses, the ACD were automatically calculated by the system [13]. The ACW and lens vault (LV) were measured manually, ACW was the distance between the two scleral spurs, and LV was defined as the distance between the anterior surface of the crystalline lens and the spur-to-spur line (Figure S1, in Supplementary Material available online at https://doi.org/10.1155/2017/6762047). The final ACD and LV were calculated as mean of the two values obtained from the horizontal and vertical images. The ACW determined from the horizontal scan was termed ACW-H and the ACW determined from the vertical scan was termed ACW-V. Eyes with any motion artefacts during OCT scanning or eyes in which the anterior chamber was partially blocked by the upper and lower limbus were excluded from the analyses. Any subjects that present angle closure on OCT images, which was defined as closed on an OCT image if contact was visible between the peripheral iris and any part of the angle wall anterior to the scleral spur, were also excluded [14].

### 2.3. Repeatability and Reproducibility

For the first 20 eyes, ACV was measured using 8 or 128 radial slices and the results were compared. These eyes were included in analyses of repeatability and reproducibility. Two series of scans were recorded in a single visit. Three days later, another series of scans was recorded. For manual measurement of ACW, ACD, and LV, the intraobserver repeatability was evaluated by one observer who measured the ACD, LV, ACW-H, and ACW-V twice. Interobserver reproducibility was determined by two observers who measured ACD, LV, ACW-H, and ACW-V independently. Intraclass correlation (ICC) coefficients and Bland–Altman plots were used to assess the reliability, repeatability, and reproducibility of the measurements. Values of 0.81–1.00 for ICC indicate almost perfect agreement and values of <0.40 indicate poor to fair agreement.

### 2.4. Statistical Analysis

Only data obtained for the right eyes were used. All analyses were performed using SPSS software version 16.0 (SPSS Inc., Chicago, USA) and MedCalc software version 11.4 (MedCalc Software, Ostend, Belgium). The level of significance was set at *P* < 0.05. Data are presented as the mean ± standard deviation (SD). Univariate and multivariate linear regression modelling analyses were performed with ACV as the dependent variable, while other ocular and general variables were included as independent variables. Stepwise multiple linear regression analyses were used to determine the contribution of each variable to the overall model, as represented by the partial* R*^2^. An* R*^2^ close to 1 indicates perfect prediction and* R*^2^ close to 0 indicates no predictive value. The associations between ACV and ocular parameters and age were also determined after dividing subjects into age-groups (by decade) to determine the annual reductions in anterior segment parameters with age using linear regression analysis. Student's *t*-test was used to compare the vertical and horizontal ACW, ACD, and LV.

## 3. Results

Six hundred and forty-seven eyes of 334 adult healthy Chinese subjects were enrolled in this study. Twenty-one eyes were excluded due to the poor-quality images (the superior limbi of fifteen eyes were covered by upper eyelids and six eyes had motion artefact), and no one was found to have angle closure on OCT images. As a result, 313 eyes of 313 healthy Chinese adults were included in the final analysis, and in 56 scans (21 eyes) the cornea and iris boundaries need to be manually adjusted. The subjects comprised 125 males and 188 females. The mean ± SD of age was 36.58 ± 9.51 years (range, 18–65 years).

The ICC for ACV calculated using 8 and 128 radial slices was 0.99. The ICCs of ACV, PD, and IV were high (all > 0.95) for both intravisit repeatability and for intervisit reproducibility. The ICCs of LV, ACD, ACW-H, and ACW-V were also high (all > 0.93) for both intraobserver repeatability and interobserver reproducibility. The Bland–Altman plots also showed good reliability in terms of ACV (Figure S2).

**Figure 1:**
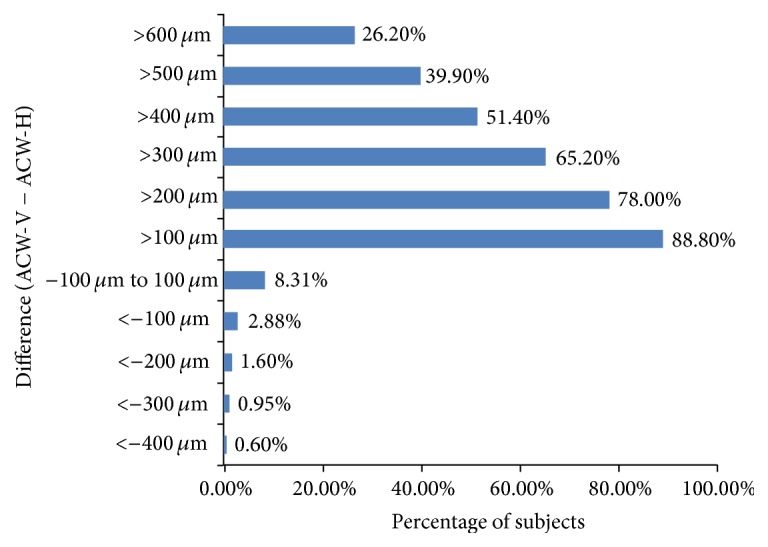
Differences in the anterior chamber width measured on horizontal and vertical scans.

The mean ACV and other anterior parameters in all 313 eyes are summarized in Table 1. A significant difference was found between ACW-H and ACW-V (*P* < 0.05). ACW-V was greater than ACW-H in 94.6% of eyes and the mean difference was 390 *μ*m. The difference between ACW-V and ACW-H was >400 *μ*m in 51.4% of eyes (Figure 1). There were no differences in ACD (*P* = 0.189) or LV (*P* = 0.295) between the horizontal and vertical scans.

Univariate analyses revealed that ACV was correlated with most of the variables, except for IOP. ACD had the greatest* R*^2^ value (0.853), followed by LV (0.508), AL (0.366), ACW-H (0.288), and ACW-V (0.235). Compared with men, women had smaller ACV (all* P* ≤ 0.001; Table 2). Multivariable analyses showed that ACV was positively associated with ACD, ACW-H, ACW-V, and AL (*P* < 0.05) and negatively correlated with IV and LV (*P* ≤ 0.001) (Table 2). The stepwise multiple linear regression model found that ACD explained 85.3% of the variability in ACV (partial* R*^2^ = 0.853; *P* < 0.001; Table 3). According to this model, for each 100-*μ*m increase in ACD, ACV increases by 6.60 mm^3^ (95% confidence interval 59.121–72.918 mm^3^; *P* < 0.001).

**Figure 2:**
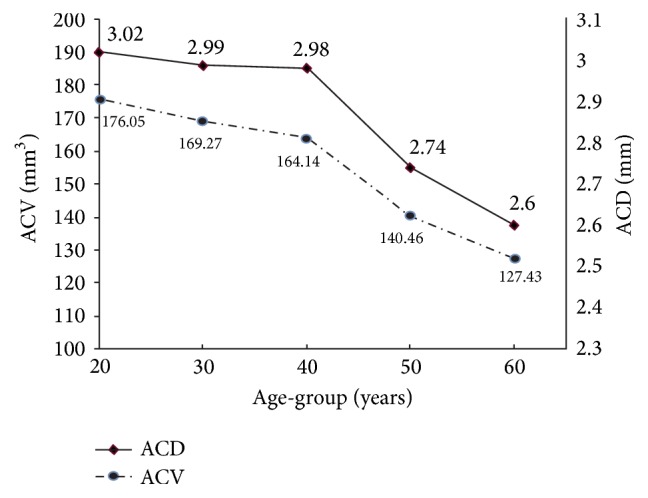
Relative reductions in anterior chamber volume (ACV) and depth (ACD) in different age-groups. Reducing rates in ACV and ACD were greatest in subjects aged 40–50 years.

Linear regression analysis showed that ACV, ACD, ACW-H, ACW-V, PD, and LV were closely correlated with age (all *P* < 0.05) but IV was not (*P* = 0.566) (Table 4). The relative change in ACV between each 10-year age-group was fastest in subjects aged 41–50 years. Similar tendencies were also observed for ACD (Table 5, Figure 2), but not for ACW-H, ACW-V, PD, and LV.

## 4. Discussion

In this study, SS-OCT was used to measure ACV and other anterior segment parameters in a relatively large group of healthy Chinese adults. The anterior segment parameters, including ACV, were successfully measured in these subjects. We found a rather large and statistically significant difference between ACW-H and ACW-V, which suggests that ACV should be calculated using more than one OCT scan. This might also be important in the measurement of volumes of the anterior or posterior segments. ACV was correlated with most of the anterior segment parameters, and its greatest association was with ACD, which explained about 85% of the variability of ACV.

Several studies have measured ACV and other anterior segment parameters in healthy eyes using different methods [15–17]. Some of these studies used indirect methods, including Scheimpflug cameras (e.g., Pentacam and Galilei Dual-Scheimpflug analyzers) [15, 17]. OCT represents a more direct method for measuring these parameters [3, 10, 11]. Unfortunately, because of the limitations of the equipment used, most of the earlier studies used only one OCT scan line to measure anterior chamber parameters [3, 10, 11]. As the anterior chamber is not part of a regular sphere, differences in ACW-H and ACW-V have been reported [18, 19], but the results varied between earlier studies. For example, Rondeau et al. [20] reported that ACW-H was greater than ACW-V while Yan et al. [18] reported that ACW-V was greater than ACW-H. Our analyses indicated that ACW-V was greater than ACW-H in 94.6% of the eyes and, in 51.4% of the eyes, the difference was >400 *μ*m. The differences in the results of these studies could be explained by differences in the methods used. Rondeau et al. used an ultrasound system and the subjects were examined in the supine position. In contrast, Yan et al. and ourselves used a noncontact OCT system with the subjects in a seated position. These different systems and the effects of gravity might account for the larger ACW-V in the seated position. Nevertheless, the mean difference between the ACW-V and ACW-H was 390 *μ*m. Therefore, considerable deviation might exist between the actual ACV and ACV measured on a single OCT scan. This deviation could be reduced by using more scan lines. The SS-OCT system used in our study acquires 128 scans in 2.4 s, and ACV could be precisely calculated by using all 128 scans. However, this is time-consuming in terms of the software calculations and the need to manually check 128 scans to confirm that the software correctly detected the location of the boundaries. In routine clinical practice, the software is also able to calculate ACV by using eight scans. In the present study, we observed excellent consistency in ACV measured using 8 and 128 scans (ICC 0.99).

The ACV (mean ± SD) in our study was 153.83 ± 32.42 mm^3^, which was within the range of 142.5–185.5 mm^3^ in prior reports [10, 15]. ACV was also closely correlated with most of the anterior segment parameters (Table 2). Hashemi et al. [21] previously reported that ACV was correlated with ACD and lens thickness. Wang et al. [3] reported that ACV was correlated with ACD, ACW, and iris area. Our results are similar to those of these earlier studies. Furthermore, stepwise multiple linear regression revealed that ACD is a major determinant of ACV and explained about 85% of the variability of ACV.

ACV and most of the anterior segment parameters, except for IV, were strongly correlated with age. Variations in ACV with age and gender have been reported. For example, Huang et al. [10] and Jóhannesson et al. [16] reported that ACV decreased with age. This tendency was also consistent with the notion that PACG, which is caused by occlusion of the drainage angle, predominantly affects elderly individuals [22, 23]. We also found that the decreasing rates in ACV and ACD, but not ACW or LV, were most prominent in subjects aged 41–50 years. Formerly, Hashemi et al. [21] also examined the relative reduction in ACV between different age-groups. Their results indicate that the reducing rate in ACV was greatest in subjects aged 40–60 years. The similar tendencies in ACV and ACD were consistent with the finding that ACD explains the greatest variability in ACV.

Decreases in ACD with age have been reported, and thickening or anterior positional movement of the crystal lens might contribute to the decreases in ACD [24, 25]. The close correlation between LV and ACV found this time was in accordance with these. Praveen et al. [26] reported that the lens thickness increased markedly in subjects aged 41–50 years, and this might account for the greater reductions in ACV and ACD in subjects aged 41–50 years in our study. A decrease in ACW with age was also found in our study. Although the reason is unclear, it might be explained as follows. Sheppard and Davies [27] reported that the ciliary muscle becomes thicker with age, and they also observed an anterior-interior shift in the ciliary muscle mass. The thickening of 3 *μ*m/year and anterior-interior shift of 4 *μ*m/year reported in that study are consistent with the reduction in ACW of 0.006 mm/year (6 *μ*m/year) observed in our study if we consider the changes in ciliary muscles on both sides of the eye. Although the thickness of the trabecular meshwork also increases with age [28], this change is relatively minor compared with the reduction in ACW of 6 *μ*m/year. The decreasing of ACD and that of ACW with aging could all contribute to the negative correlation between ACV and age.

The current study was limited by its cross-sectional design and the enrolment of only Chinese adults. Therefore, our results should be verified by other researchers.

In conclusion, by using SS-OCT, we were able to measure ACV and other anterior segment parameters in a relatively large group of healthy Chinese adults. The relatively large difference between ACW-H and ACW-V suggests that ACV should be calculated using multiple OCT images. SS-OCT could provide a reliable method for monitoring the changes in ACV and other anterior segment parameters over time.

## Supplementary Material

Figure S1: Measurement of the anterior segment parameters.
Figure S2. Bland–Altman plots for the agreement in anterior chamber volume measurement.

## Figures and Tables

**Table 1:** Demographic characteristics of the subjects.

Variable	Mean ± SD	Minimum	Maximum
Age, years	36.58 ± 9.51	18	65
AL, mm	23.28 ± 0.82	21.37	24.97
IOP, mmHg	13.55 ± 2.67	7	21
ACV, mm^3^	153.78 ± 32.45	56.61	267.16
ACD, mm	2.88 ± 0.32	1.56	3.81
ACW-H, mm	11.45 ± 0.39	10.26	12.70
ACW-V, mm	11.88 ± 0.44	10.2	13.13
IV, mm^3^	34.74 ± 4.34	19.77	45.92
LV, mm	0.08 ± 0.27	−0.55	0.96
PD, mm	5.12 ± 0.98	2.56	7.88

SD = standard deviation, AL = axial length, IOP = intraocular pressure, ACV = anterior chamber volume, ACD = anterior chamber depth, ACW-H = anterior chamber width measured in the horizontal direction, ACW-V = anterior chamber width measured in the vertical direction, IV = iris volume, LV = lens vault, and PD = pupil diameter.

**Table 2:** Univariate and multivariate analyses of the associations between anterior chamber volume and ocular or general characteristics.

	Univariate analyses			Multivariable analyses	
	*β* (95% CI)	*R* ^2^	*P*	*β* (95% CI)	*P*
Age, years	−1.436 (−1.781 to −1.091)	0.178	<0.001^*∗*^	−0.085 (−0.224 to 0.053)	0.227
Gender	14.809 (7.617 to 22.002)	0.050	<0.001^*∗*^	3.337 (0.986 to 5.688)	0.033^*∗*^
AL, mm	23.92 (20.406 to 27.433)	0.366	<0.001^*∗*^	3.228 (1.111 to 5.345)	0.003^*∗*^
IOP, mmHg	−0.066 (−1.424 to 1.291)	0.000	0.924	0.274 (−0.13 to 0.579)	0.183
PD, mm	13.914 (10.582 to 17.246)	0.178	<0.001^*∗*^	0.721 (−0.555 to 1.996)	0.267
LV, mm	−86.654 (−96.177 to −77.131)	0.508	<0.001^*∗*^	−20.188 (−29.171 to 11.205)	<0.001^*∗*^
IV, mm^3^	1.425 (0.607 to 2.243)	0.036	0.001^*∗*^	−0.513 (−0.803 to −0.224)	0.001^*∗*^
ACW-H, mm	44.483 (36.685 to 52.28)	0.288	<0.001^*∗*^	19.397 (14.187 to 24.608)	<0.001^*∗*^
ACW-V, mm	17.765 (12.749 to 22.78)	0.235	<0.001^*∗*^	2.052 (0.167 to 3.937)	0.006^*∗*^
ACD (mm)	94.801 (90.415 to 99.187)	0.853	<0.001^*∗*^	65.5 (58.603 to 72.397)	<0.001^*∗*^

^*∗*^ *P* < 0.05.

Univariate and multivariable linear regression analyses were performed with ACV as the dependent variable and age, gender, AL, IOP, PD, LV, IV, and ACW as independent variables.

CI = confidence interval, AL = axial length, IOP = intraocular pressure, PD = pupil diameter, LV = lens vault, IV = iris volume, ACW-H = anterior chamber width measured in the horizontal direction, ACW-V = anterior chamber width measured in the vertical direction, and ACD = anterior chamber depth.

**Table 3:** Stepwise multiple linear regression analysis of factors associated with anterior chamber volume.

Factors included in the model	Factor	Partial *R*^2^	Model *R*^2^	*β* (95% CI)	*P*
(1)	ACD, mm	0.853	0.853	66.019 (59.121 to 72.918)	<0.001^*∗*^
(2)	ACW-H, mm	0.037	0.890	22.394 (17.764 to 27.024)	<0.001^*∗*^
(3)	LV, mm	0.016	0.906	−23.276 (−31.707 to −14.845)	<0.001^*∗*^
(4)	AL, mm	0.003	0.909	2.707 (−0.674 to 4.739)	<0.001^*∗*^
(5)	ACW-V, mm	0.003	0.912	4.286 (1.284 to 8.58)	0.009^*∗*^
(6)	IV, mm	0.002	0.914	−0.546 (−0.828 to −0.263)	0.009^*∗*^

^*∗*^ *P* < 0.05.

CI = confidence interval, ACD = anterior chamber depth, ACW-H = anterior chamber width measured in the horizontal direction, LV = lens vault, AL = axial length, ACW-V = anterior chamber width measured in the vertical direction, and IV = iris volume.

**Table 4:** Associations between ocular parameters and age.

	*β*	*P*
ACV	−1.436	<0.001^*∗*^
ACD	−0.021	<0.001^*∗*^
ACW-H	−0.005	0.043^*∗*^
ACW-V	−0.006	0.017^*∗*^
IV	0.015	0.566
PD	−0.04	<0.001^*∗*^
LV	0.013	<0.001^*∗*^

^*∗*^ *P* < 0.05.

ACV = anterior chamber volume, ACD = anterior chamber depth, ACW-H = anterior chamber width measured in the horizontal direction, ACW-V = anterior chamber width measured in the vertical direction, IV = iris volume, PD = pupil diameter, and LV = lens vault.

**Table 5:** Annual reductions in anterior segment parameters according to the age of subjects.

Age-group, years	ACV		ACD		ACW-H		ACW-V		LV		PD	
	mm^3^/year	%	mm/year	%	mm/year	%	mm/year	%	mm/year	%	mm/year	%
21–30	−0.887	0.45	−0.019	0.45	−0.003	0.02	−0.005	0.04	0.02	3.6	−0.092	1.1
31–40	−1.547	0.68	−0.018	0.49	−0.008	0.07	−0.007	0.06	0.012	9.8	−0.069	0.8
41–50	−3.019	1.12	−0.029	0.79	−0.005	0.04	−0.004	0.03	0.023	5.4	−0.076	0.9
51–60	−2.244	0.98	−0.022	0.82	−0.031	0.22	−0.04	0.28	0.005	1.2	−0.004	0.1

Results are presented as the mean.

ACV = anterior chamber volume, ACD = anterior chamber depth, ACW-H = anterior chamber width measured in the horizontal direction, ACW-V = anterior chamber width measured in the vertical direction, LV = lens vault, and PD = pupil diameter.
