# Supplementary material for: Measurement of and Factors Associated with the Anterior Chamber Volume in Healthy Chinese Adults
Source: J Ophthalmol. 2017 Jan 10;2017:6762047. doi: 10.1155/2017/6762047 (PMC5259661; doi:10.1155/2017/6762047)
Supplement: Supplementary file 1 — Figure S1: Measurement of the anterior segment parameters. Figure S2. Bland–Altman plots for the agreement in anterior chamber volume measurement. [file 6762047.f1.docx]

**Supplementary materials**

**Figure S1.** Measurement of the anterior segment parameters on a horizontal swept-source optic coherence tomography slice. After manually locating the angle recesses (AR1 and AR2), the points CCT-B, ACD-L, and anterior chamber depth (ACD; Endo.) were automatically placed by the system. CCT-B was located at the intersection of the posterior corneal surface and the central vertical line of the anterior chamber. ACD-L was located at the intersection of the anterior lens capsule and the central vertical line of the anterior chamber. The ACD (ACD [Endo.]) was defined as the distance between CCT-B and ACD-L. The anterior chamber width (ACW) was calculated as the distance between the two scleral spurs (SP1 and SP2). We manually relocated ACD-L to the intersection between the central vertical line and the line between SP1 and SP2. A second ACD (Endo) was calculated as the distance between CCT-B and ACD-L’. The lens vault was calculated the distance between ACD-L and ACD-L’ (i.e., the distance between CCT-B and ACD-L’ − the distance between CCT-B and ACD-L).


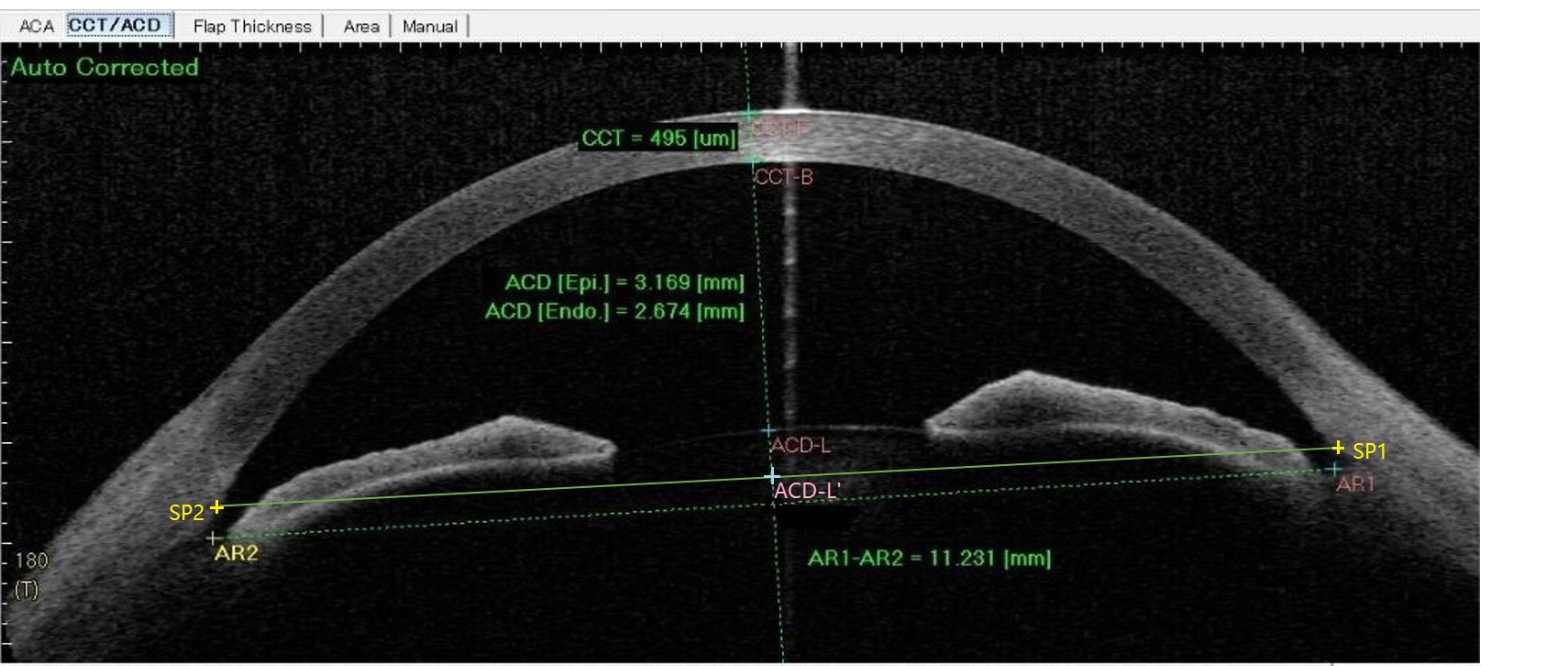


**Figure S2.** Bland–Altman plots for the agreement in anterior chamber volumes (ACV) calculated using 8 or 128 slices (A), intra-visit repeatability of ACV (B), and inter-visit reproducibility of ACV (C). ACV8 = ACV determined from 8 slices, ACV128 = anterior chamber volume determined from 128 slices, ACVs1 and ACVs2 = ACV calculated from the two series of scans acquired on the same visit, ACVd1 and ACVd2 = ACV calculated from the two series of scans acquired 3 days apart, SD = standard deviation.

A

B

C
